# Harnessing translational research in wheat for climate resilience

**DOI:** 10.1093/jxb/erab256

**Published:** 2021-06-17

**Authors:** Matthew P Reynolds, Janet M Lewis, Karim Ammar, Bhoja R Basnet, Leonardo Crespo-Herrera, José Crossa, Kanwarpal S Dhugga, Susanne Dreisigacker, Philomin Juliana, Hannes Karwat, Masahiro Kishii, Margaret R Krause, Peter Langridge, Azam Lashkari, Suchismita Mondal, Thomas Payne, Diego Pequeno, Francisco Pinto, Carolina Sansaloni, Urs Schulthess, Ravi P Singh, Kai Sonder, Sivakumar Sukumaran, Wei Xiong, Hans J Braun

**Affiliations:** 1 International Maize and Wheat Improvement Center (CIMMYT), Texcoco, Mexico; 2 School of Agriculture, Food and Wine, University of Adelaide, Waite Campus, PMB1, Glen Osmond SA 5064, Australia; 3 Wheat Initiative, Julius Kühn-Institute, Königin-Luise-Str. 19, 14195 Berlin, Germany; 4 CIMMYT-Henan Collaborative Innovation Center, Henan Agricultural University, Zhengzhou, 450002, PR China; 5 The University of Queensland, Australia

**Keywords:** Abiotic, big data, breeding, climate resilience, environment, genetic resources, genomics, international collaboration, phenomics, physiology

## Abstract

Despite being the world’s most widely grown crop, research investments in wheat (*Triticum aestivum* and *Triticum durum*) fall behind those in other staple crops. Current yield gains will not meet 2050 needs, and climate stresses compound this challenge. However, there is good evidence that heat and drought resilience can be boosted through translating promising ideas into novel breeding technologies using powerful new tools in genetics and remote sensing, for example. Such technologies can also be applied to identify climate resilience traits from among the vast and largely untapped reserve of wheat genetic resources in collections worldwide. This review describes multi-pronged research opportunities at the focus of the Heat and Drought Wheat Improvement Consortium (coordinated by CIMMYT), which together create a pipeline to boost heat and drought resilience, specifically: improving crop design targets using big data approaches; developing phenomic tools for field-based screening and research; applying genomic technologies to elucidate the bases of climate resilience traits; and applying these outputs in developing next-generation breeding methods. The global impact of these outputs will be validated through the International Wheat Improvement Network, a global germplasm development and testing system that contributes key productivity traits to approximately half of the global wheat-growing area.

## Teaser

This review describes the scientific basis of a research and breeding pathway designed to boost genetic gains in wheat under drought and heat stress.

## Introduction

Wheat (*Triticum aestivum* and *Triticum durum*) is a key pillar of food security, being the most widely grown crop worldwide and constituting 20% of all human calories and protein (Shiferaw *et al.*, 2013). However, environmental factors threaten production of wheat and many other field-based crops. The past decade has been identified as the warmest on record (NOAA National Centers for Environmental Information, 2020), and the Intergovernmental Panel on Climate Change (IPCC) predicts that by 2050, global average temperature may rise as much as 2 °C over pre-industry levels (and nearly 5 °C by 2100), while historic rainfall patterns are disrupted (Field *et al.*, 2014). Such changes are now affecting food security with ‘high confidence’ (IPCC, 2018, 2019; FAO *et al.*, 2019). Furthermore, climate-based reductions in agricultural productivity have been an important factor in human migrations (Glaser *et al.*, 2017; Falco *et al.*, 2019; FAO and WFP, 2019) and are predicted to be a critical driver in the future (Rigaud *et al.*, 2018). Recent research has highlighted risks of simultaneous crop failures due to heat and/or drought in ‘breadbaskets’ across the globe (Maynard, 2015; Sarhadi *et al.*, 2018; Gaupp *et al.*, 2020; Kornhuber *et al.*, 2020), and extremes in temperatures and precipitation (including drought) are already attributed with 40% of interannual production variability in wheat (Zampieri *et al.*, 2017). Independent of changes in water stress, with each 1 °C increase in average temperature, global wheat production is predicted to decrease by 6% (Liu *et al.*, 2016; Zhao *et al.*, 2017). Although some research and modelling studies indicate that rising levels of atmospheric CO_2_ at least partially offset harmful effects of heat and drought stress, data are far from consistent (Sreeman *et al.*, 2018; Tcherkez *et al.*, 2020; Zheng *et al.*, 2020). Furthermore, the models neglect harmful effects of rising night temperature, heat shocks, unstable rainfall patterns, and nutritional factors, for which there is no evidence of amelioration by elevated CO_2_.

**Fig. 1.**
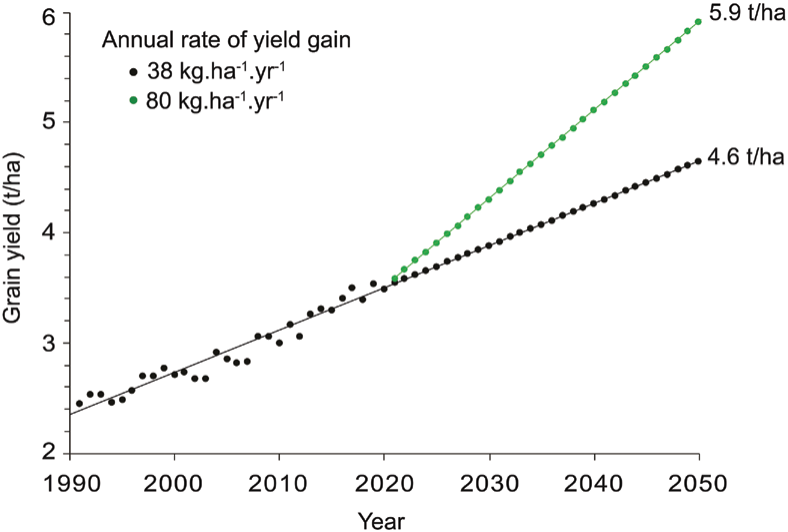
Historical and future projected grain yield for wheat. Historical data from the previous 30 years were used. A similar yield trend was observed with 60 previous years of data (Wulff and Dhugga, 2018). The average annual yield increase over the last 30 years has been 38 kg ha^−1^ year^−1^. Extrapolation with the current annual rate of gain to 2050 leads to 4.6 t ha^−1^ grain yield, which is an increase of a little over 30% above the 2020 level of 3.5 t ha^−1^ (black). Projected need (green) from a growing and increasingly affluent population is ~1.3 billion Mt by 2050 (Ray *et al.*, 2013), which, in order to be met, requires an annual rate of gain of 80 kg ha^−1^ year^−1^ for a yield of 5.9 t ha^−1^. Updated data (13 August 2020) for wheat production were downloaded from United States Department of Agriculture–Economic Research Service site (https://www.ers.usda.gov/data-products/wheat-data/).

However, timely interventions can mitigate many of the negative effects of unfavourable climates. In a meta-analysis of 1700 published simulations to evaluate yield impacts of climate change and benefits of adaptation, the authors concluded that cultivar improvement was the most effective method for adaptation (Challinor *et al.*, 2014; Beacham *et al.*, 2018). While genetic gains must increase substantially to match the many challenges that wheat and other crops face (Fig. 1), incorporating technological advancements into plant breeding operations can enable such gains, though investment and strategic implementation are critical. Though investments in wheat in recent years have fallen behind other staple crops (e.g. 4-fold more is invested in maize R&D) (Manners and van Etten, 2018), with well-coordinated investment and strategic planning, continual developments in plant science and genetics can be harnessed to breeding efforts through translational research, fuelled by the ever more powerful tools of genomics, phenomics, and informatics (Jha *et al.*, 2014; Mathan *et al.*, 2016; Araus *et al.*, 2018; Govindaraj *et al.*, 2018; Hu *et al.*, 2018; Lamaoui *et al.*, 2018; Sreeman *et al.*, 2018; Varshney *et al.*, 2020). In addition to helping identify and combine beneficial alleles within extant genepools, such technologies can be applied to the discovery of novel traits and alleles from among the vast and relatively untapped reserve of wheat genetic resources, of which an estimated 0.8 million accessions exist across >80 collections worldwide, though some redundancy of accessions across collections is expected (International Maize and Wheat Improvement Center, 2007; Singh *et al.*, 2019).

**Fig. 2.**
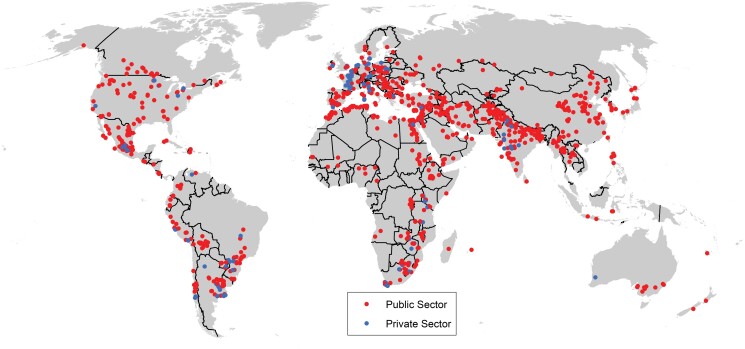
Public and private breeding programmes that have received germplasm under the International Wheat Improvement Network.

**Fig. 3.**
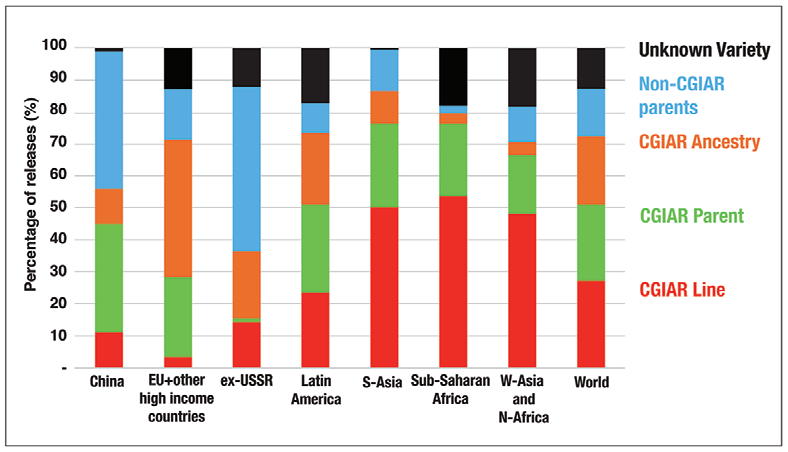
Spring bread wheat released by region and origin through the IWIN, 1994–2014 (Lantican *et al.*, 2016). Adapted under CC BY-NC.

Systematic breeding of wheat goes back >100 years, with key advances in breeding approaches rapidly transferred around the world (Lupton, 1987). Internationally, wheat breeding has been dominated by the public sector and this has been supported through the extensive exchange of germplasm and advances (Fig. 2). Foremost among these exchanges is the International Wheat Improvement Network (IWIN)—a legacy of the Green Revolution—which continues to provide advanced breeding lines to wheat breeders worldwide (Fig. 2; Reynolds *et al.*, 2017*a*), now under the CGIAR Research Program on Wheat https://wheat.org/. The result is that there are strong links in pedigrees of wheat varieties from very diverse breeding programmes. The continued improvement of wheat is greatly influenced by the maintenance of such free exchange. The role of CIMMYT (International Maize and Wheat Improvement Center) and the establishment of the IWIN has been particularly important in promoting the sharing and evaluation of germplasm. Impacts of the IWIN are apparent from the fact that >50% of all cultivars released, globally, contain IWIN germplasm in their pedigree, while many advanced lines are released directly as cultivars (Fig. 3, spring bread wheat; see Lantican *et al.*, 2016 for complementary figures regarding winter/facultative bread wheat and spring durum wheat). The IWIN encompasses public and private breeding programmes and research institutes from almost all wheat-producing countries, most of which share genetic resources, expertise, and data. IWIN’s success is underpinned by its highly strategic breeding goals coordinated by CIMMYT, including yield gains and stability, resistance to the most prevalent diseases, targeted end-use quality, and adaptation to abiotic stresses (Braun *et al.*, 2010). As a result of this unique collaboration, returns on public investment are extremely high (estimated recently at ~100:1; Lantican *et al.*, 2016), genetic gains remain steady for spring wheat (Crespo-Herrera *et al.*, 2017, 2018), potential disease pandemics such as stem rust have been avoided (Singh *et al.*, 2011), while natural ecosystems have been protected from cultivation due to food sufficiency (Stevenson *et al.*, 2013). While the historic basis for investment in the IWIN has largely focused on less developed countries, most developed countries have also benefited. For example, the majority of wheat grown in Australia derives from the IWIN (Brennan and Quade, 2006), as do sources of disease resistance for many lines released in the USA where >50% of the wheat acreage is sown to CIMMYT-related varieties (Lantican *et al.*, 2016).

**Fig. 4.**
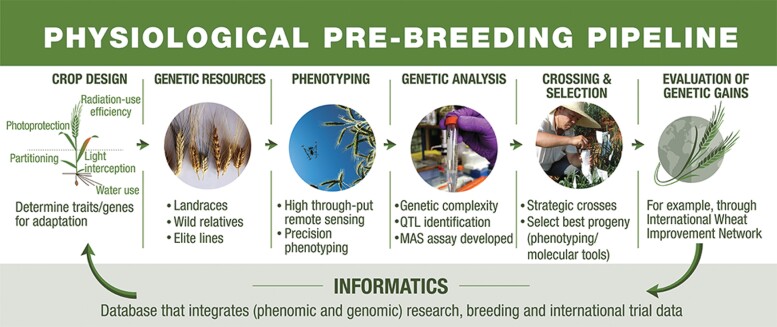
Main research steps involved in translating promising technologies into genetic gains (graphical abstract, adapted from Reynolds and Langridge, 2016). Reprinted under licence CC BY-NC-ND.

However, given the many new challenges that wheat breeders must face, wheat improvement efforts need a significant boost. It is apparent from the scientific literature that many promising plant discoveries are not translated into breeding technologies. One of the key reasons is the bottleneck that exists between discovery research and breeding *per se*, thereby limiting the societal value of enormous investments in the former (Reynolds *et al.*, 2019). Herein are outlined a number of approaches that can bridge this gap based on combining recent research advances with tried and tested breeding methods. The research objectives discussed include: improving definitions of target environments using deep learning of big data sets to better design and deploy cultivars encompassing the appropriate adaptive traits; adapting phenomic and genomic technologies to multiple uses—from accessing untapped genetic resources, through parental and progeny screening to gene discovery; accelerating genetic gains under a harsher climate through refinement and adoption of new breeding techniques (Fig. 4); and crowd-sourcing novel ideas and technologies for testing and validation in a realistic breeding context.

Linking these translational research activities to the IWIN can generate considerable scale-out, aided by access to cutting-edge germplasm, extensive databases relevant to target environments, and to the actual field environments currently used to achieve genetic gains worldwide through the IWIN. Crowd-sourcing of novel ideas and technologies can help facilitate engagement with a range of international collaborators, including those of the Heat and Drought Wheat Improvement Consortium (HeDWIC, http://www.hedwic.org/). The types of research synergies generated by such research platforms has already been demonstrated under the International Wheat Yield Partnership (IWYP, https://iwyp.org/).

**Fig. 5.**
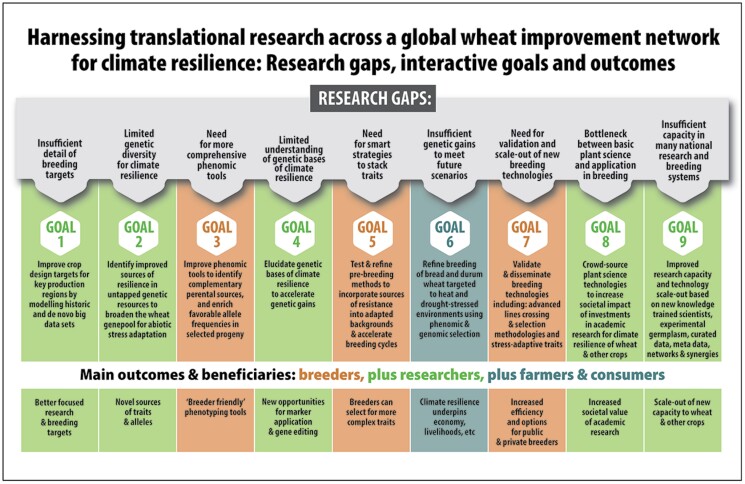
Harnessing research across a global wheat improvement network for climate resilience: research gaps, interactive goals, and outcomes.

This review proposes ways to boost benefit from new plant science technologies by strategically harnessing promising translational research to successful breeding programmes, thereby accelerating delivery of climate-resilient wheat. The ideas presented in this review form part of a new collaborative initiative linking a number of networks to a translational research and breeding pipeline led by the HeDWIC—which is coordinated by CIMMYT—that addresses nine specific research-related gaps (Fig. 5), which together are expected to boost genetic gains in wheat under drought and heat stress. This review will present the scientific background related to research goals related to gaps 1–5 and briefly summarize how they will complement goals 6–9 (Fig. 5).

## Improving crop design targets for key production regions by modelling historic and *de novo* big data sets

Breeders are faced with the problem of ensuring their varieties meet the local needs of growers while also providing sufficiently broad adaptation to justify the investment and scale of their selection programmes. Consequently, breeders must find a balance between adaptation and yield stability for a particular target population of environments, while ensuring new varieties will be adapted to seasonal effects within the target population of environments. Therefore, one of the most difficult challenges in crop improvement is to select, under a restricted range of breeding environments, outstanding new cultivars that will be adapted to a much wider range of environments (including seasonal effects) due to the phenomenon of genotype by environment interaction (GEI). The main causes of GEI, assuming pests and diseases are controlled genetically or through husbandry, are related to the climate, the soil, and crop management. While the latter is controllable by farmers, the effects of the climate and soil are much less so. In order to improve the efficacy of breeding strategies, the principal causes of GEI need to be better understood, as outlined in this section.

### Refining breeding targets for key production areas by trialling genetically diverse modern germplasm under diverse stress profiles

While it is recognized that different heat and drought profiles exist across the range of wheat cropping systems (Braun *et al.*, 2010), many wheat breeding programmes do not have the capacity to proactively screen for heat and drought resistance and/or may apply quite generic heat and drought stress treatments. Furthermore, changing weather patterns are making the prediction of future environments critical to long-term breeding success. It has also been shown that several collateral factors, such as soil micronutrient deficiencies or toxicity, and biotic stresses, if not properly identified, can seriously confound research attempting to identify the physiological and genetic bases of stress adaptation, as well as diminish expected genetic gains from breeding (Bagci *et al.*, 2007; Mathews *et al.*, 2011). Therefore, elucidation of different stress profiles that wheat must adapt to, in terms of its phenological development and adaptive traits at key target locations, is a valuable research goal. At the same time, collateral constraints to yield can be considered.

The elucidation of stress profiles along with collateral constraints can be addressed by trialling representative heat- and drought-adapted genotypes under different abiotic stress profiles at a range of international target sites, as well as locations that represent a range of predicted environments (hereafter referred to as ‘future climate analogue sites’) (Ramírez-Villegas *et al.*, 2011; Opare *et al.*, 2018). Agronomic, phenological, and physiological data, plus metadata (weather, soils, and crop management), can be used to show which traits are most sensitive to GEI and which environmental factors are driving the interactions (Reynolds *et al.*, 2004). Molecular markers associated with adaptation to specific stress profiles as well as main effects of heat and/or drought can be identified using this approach (Messmer *et al.*, 2009). As with any objective of this magnitude, the resources to encompass enough sites to represent millions of hectares of cropping systems is a challenge, and the next section will address approaches to achieve this using historical IWIN data, though restricted to yield and heading date, and using reconstructed weather data. Targeted trialling can go deeper in terms of precise phenology, key physiological traits, and precise environmental characterization, though at a restricted range of sites, depending on resources. Nonetheless, outputs from this exercise can be used to update data collection protocols, such that the underlying causes of GEI can be addressed on a much larger scale.

### Planning for future climate scenarios in key production areas by modelling big data sets

Over approximately four decades, the IWIN has amassed millions of yield and other agronomic data points from nurseries grown by breeders in >90 countries (Fig. 2; Reynolds *et al.*, 2017*a*). Beyond some attempts to assess genetic gains in the CIMMYT wheat programme (Sharma *et al.*, 2012; Gourdji *et al.*, 2013; Crespo-Herrera *et al.*, 2017, 2018; Gerard *et al.*, 2020), relatively few of these data have been explored, due in part to a lack of metadata, such as temperature, radiation, and other environmental information associated with each testing nursery. However, reconstructed hourly weather data are now freely accessible (e.g. ERA5, https://www.ecmwf.int/en/forecasts/datasets/reanalysis-datasets/era5). Combined with the extensive pedigree relationship information recorded for IWIN accessions, the capacity to cheaply genotype those accessions, and the availability of the reference genome for wheat (International Wheat Genome Sequencing Consortium, 2018), considerable opportunities now exist to model GEI and plan for future climate scenarios.

The IWIN data sets, which represent some of the most extensive publicly available records characterizing balanced multienvironment trials conducted across many locations and environmental conditions (Table 1), present a unique opportunity to explore the various dimensions of GEI. For example, heading date is the most widely recorded trait within the IWIN data sets after grain yield, opening up the possibility to conduct the largest evaluation to date of the effects of the constellation of flowering time genes in wheat (*Ppd*, *Vrn*, and *Eps*), which underpin the reproductive biology and determination of yield under different heat and drought profiles (Zheng *et al.*, 2013). In addition, modelling approaches that incorporate environmental and genomic data (Heslot *et al.*, 2014; Jarquín *et al.*, 2014; Crossa *et al.*, 2017; Li *et al.*, 2018; Messina *et al.*, 2018; Montesinos-López *et al.*, 2018*a*) can be leveraged to identify genetic loci associated with phenotypic stability and response to abiotic stress, and to predict crop performance in untested environments and in future years.

**Table 1.** International nurseries annually distributed by the CIMMYT within the International Wheat Improvement Network (IWIN)

Nursery type	Trial/Nursery	Abbreviation	Target megaenvironment(s) (MEs)^*a*^	Grain colour	BW/DW^*b*^
**Yield trials**	Elite Spring Wheat Yield Trial	ESWYT	ME1	White	BW
	Harvest Plus Yield Trial	HPYT	ME1	White	BW
	Heat Tolerant Wheat Yield Trial	HTWYT	ME5	White	BW
	High Rainfall Wheat Yield Trial	HRWYT	ME2	Red	BW
	International Durum Yield Nursery	IDYN	ME1, ME4, ME5		DW
	Semi Arid Wheat Yield Trial	SAWYT	ME4	White	BW
	South Asia Bread Wheat Genomic Prediction Yield Trial	SABWGPYT	ME1, ME4, ME5	White	BW
	Wheat Yield Consortium Yield Trial	WYCYT			BW
**Observation**	International Bread Wheat Screening Nursery	IBWSN	ME1, ME4, ME5	White	BW
	High Rainfall Wheat Screening Nursery	HRWSN	ME2	Red	BW
	International Durum Screening Nursery	IDSN	ME1, ME4, ME5		DW
	Semi Arid Wheat Screening Nursery	SAWSN	ME4	White	BW
**Trait specific**	Fusarium Head Blight Screening Nursery	FHBSN			BW
	Harvest Plus South Asia Screening Nursery	HPAN	ME1, ME4, ME5	White	BW
	Heat Tolerance Screening Nursery	HTSN	ME5	White/red	BW
	Helminthosporium Leaf Blight Screening Nursery	HLBSN			
	International Septoria Observation Nursery	ISEPTON			BW
	Karnal Bunt Screening Nursery	KBSN		White	BW
	Stem Rust Resistance Screening Nursery	SRRSN		White/red	BW
	Stress Adaptive Trait Yield Nursery	SATYN			BW

^*a*^ See Gbegbelegbe *et al.* (2017) for a description and maps related to mega-environments.

^*b*^ BW, bread wheat; DW, durum wheat

The wide spatiotemporal variability in the IWIN data sets also facilitates strategic planning for future growing conditions impacted by climate change. For example, by leveraging empirical performance and environment data over time, it will be possible to evaluate the stability of CIMMYT’s system of mega-environments, which were originally defined broadly according to plant, disease, edaphic, and climate characteristics (Braun *et al.*, 2010). This exercise would also inform the development of a more dynamic strategy to geographically characterize wheat production regions that can account for year to year variation, which the current system ignores (Trethowan *et al.*, 2005; Hyman *et al.*, 2013). In addition, wheat performance under diverse heat and drought profiles has been sampled widely within the IWIN data sets. Analyses of such data sets can enable the determination of critical heat and drought thresholds at specific growth stages and provide insight into how wheat germplasm must be adapted for future climate analogues (Zampieri *et al.*, 2018). Those IWIN accessions least affected by heat and drought stress or other weather anomalies may be useful as parent material for breeding programmes or for use in additional experiments to elucidate the underlying genetic and physiological mechanisms conferring climate resilience.

Emerging analytics methods such as deep learning (DL) (Montesinos-López *et al.*, 2018*a*, *b*, 2019), which is a type of machine learning approach and a subfield of artificial intelligence (AI), may be useful to address the considerable volume of data and metadata produced by the IWIN. Considerable empirical evidence exists demonstrating the power of DL as a tool for developing AI systems, products, devices, apps, etc. Leveraging advances made in DL within the context of the IWIN offers a unique opportunity to link genomic, environmental, and phenotypic information at an immense scale.

### Crop simulation to predict optimal phenology and growth pattern of wheat under different heat and drought stress profiles

Crop growth, development, and grain yield are results of the cumulative influence of complex interactions between environmental factors and crop traits (Reynolds *et al.*, 2020). Crop simulation can help elucidate the basis of GEI, thereby helping predict the optimal phenology and growth pattern of wheat under different heat and drought stress profiles at different scales using a multimodel ensemble point and gridded simulations (Wallach *et al.*, 2021).

A new global gridded wheat modelling capacity developed in a consortium led by the University of Florida, CIMMYT, and the International Food Policy Research Institute (IFPRI) (Gbegbelegbe *et al.*, 2017) has been extended to a multimodel capacity using three DSSAT wheat models—CROPSIM-CERES, CROPSIM, and NWheat (Hernandez-Ochoa *et al.*, 2019; Pequeno *et al.*, 2021). Crop simulation models are embedded within the Mink system, which is a global scale gridded simulation platform for the use of economic models of agriculture at a global scale (Robertson, 2017). Recently this system is also running in high-performance computer clusters of CIMMYT to perform global future climate change scenarios studies using 30 years of current (1980–2010) and future climate scenarios (2040–2070) (Pequeno *et al.*, 2021).

A multicrop model and multiglobal climate model ensemble in a gridded format across wheat-growing regions can be used to: (i) collate high-quality drought and heat years data to calibrate and validate model simulation response to heat and drought, with particular emphasis on any yield reductions associated with the impact of high temperature on heading date and length of the grain-filling period, and the impact of drought affecting crop assimilation and expansive growth processes; (ii) run historical simulations (1980–2010) with the best combination of traits for heat and drought environments to maximize average grain yield including optimized phenological length, drought escape (Lopes *et al.*, 2018), and early vigour as adaptation to terminal drought (Asseng *et al.*, 2003); and (iii) run climate change effects scenarios (2040–2070) on crop growth and development to determine the best combination of traits (Asseng *et al.*, 2002; Hernandez-Ochoa *et al.*, 2019) to maximize yield under future climate (Pequeno *et al.*, 2021). Together these three activities can achieve a better definition of the range of heat and drought stress breeding targets to help focus all aspects of breeding and research, specifically: (i) the physical environment in terms of current heat and drought stress profiles and predicted future climate scenarios (including some collateral stress factors that potentially confound research and breeding outcomes); (ii) growth and developmental patterns associated with different stress profiles and their genetic bases (including factors contributing to yield stability); and (iii) key crop traits pivotal to adaptation, which will also be estimated through crop simulation models. Curated big data and metadata sets will also be available to the crop modelling community.

### Identify improved sources of climate resilience in untapped genetic resources to broaden the wheat genepool in terms of abiotic stress adaptation

In wheat, there have been a number of successes mining exotic gene pools to identify novel sources of traits, mainly related to disease resistance, but including some serendipitous effects on yield (Reynolds *et al.*, 2007; Ortiz *et al.*, 2008; Lopes and Reynolds, 2011; Cossani and Reynolds, 2015). The best example has been the introduction of the short arm of rye chromosome 1 that conferred wide adaptation and high yield performance (Rajaram *et al.*, 1983). The genes responsible for this are largely unknown, but recent studies have shown that one factor is related to root architecture (Howell *et al.*, 2014, 2019). Compared with wheat wild relatives, landraces and synthetic hexaploids are much easier to cross with, while still representing novel pools of allelic diversity. Despite limited screening under heat and drought, impacts from targeted crossing with these sources have been shown (Reynolds *et al.*, 2017*b*).

**Fig. 6.**
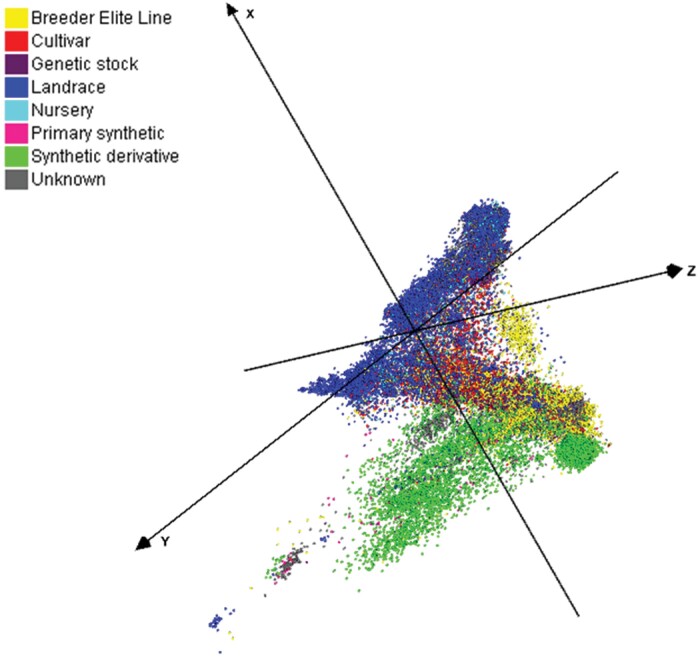
Diversity analysis of domesticated hexaploid accessions (from Sansaloni *et al.*, 2020). Multidimensional scaling plot of 56 342 domesticated hexaploid accessions with 66 067 SNP markers differentiated by biological status based on passport information (breeder elite line, landraces, cultivar, synthetic, etc.) enabling selection of research panels based on molecular diversity. Reprinted under licence CC BY.

A combination of genotypic and phenotypic profiling of genetic resource collections is a targeted approach to identify novel genetic diversity for pre-breeding and research. Approximately 0.8 million wheat genetic resource accessions are available across >80 collections globally, with >460 000 of these accessions catalogued in Genesys (https://www.genesys-pgr.org/) (International Maize and Wheat Improvement Center, 2007). The largest wheat collections identified in Genesys, currently, include the CIMMYT (>146 000 accessions), the USDA-ARS National Small Grains Germplasm Research Facility (>62 000), the International Center for Agricultural Research in Dry Areas (ICARDA, >44 000), the Australian Grains Genebank (>42 000), and the N.I. Vavilov Research Institute of Plant Industry (>35 000). The majority of these vast wheat genetic resources have never been evaluated for climate resilience attributes. As some redundancy is expected (though a thorough examination has not been made) and collections vary in types of accession (such as elite breeding germplasm, cultivars, landraces, wild relatives, lines with chromosomal introgressions, etc.) it is important to explore genetic resources in a targeted data-driven manner, such as through the selection of core sets to avoid redundancy and maximize genetic diversity (Brown, 1989; International Maize and Wheat Improvement Center, 2007; Singh *et al.*, 2019; Sansaloni *et al.*, 2020). The Seeds of Discovery initiative genotyped nearly 80 000 accessions spanning numerous types of germplasm (landraces, genetic stocks, synthetics, breeder elite lines, cultivars, etc.) from two of the largest genebanks (CIMMYT and ICARDA) using high-throughput DArTseq technology (Sansaloni *et al.*, 2011, 2020). Diversity analysis using >66 000 single nucleotide polymorphism (SNP) markers highlighted that relatively little of the genetic diversity available in landraces has been used in modern breeding, which represents a fertile ground for exploration and application in breeding (Fig. 6; Sansaloni *et al.*, 2020). Considering this, it is crucial to define and implement clear strategies to explore and use relevant genetic diversity for breeding. Linking the information from genomic profiles and the phenotypic data from field trials conducted under abiotic stresses (heat and drought) therefore offers pioneering opportunities for the development of more precise and integrative diversity panels (Reynolds *et al.*, 2015) with increased value for trait identification and allele mining. With that, we can leverage relevant diversity from the shelves of germplasm vaults into the hands of breeders.

Characterization of unadapted, wild germplasm for traits important to modern agriculture has its challenges, especially for traits controlled by many genes. For example, phenology of wild germplasm may be out of sync with locally adapted checks, making the timing of stress treatments and direct comparison with adapted germplasm problematic. There is also the possibility that constitutive stress-adaptive traits could negatively impact yield in more favourable cycles, for example high transpiration efficiency was associated with reduced stomatal conductance (Farquhar *et al.*, 1989). Nonetheless, successes have been reported for abiotic as well as biotic traits, as outlined in the next section, and there is growing interest in exploring new approaches to deploying genetic diversity in breeding programmes, such as redomestication (Langridge and Waugh, 2019).

### Introgress novel sources of heat/drought resilience from wild relatives and ancestral genomes into adapted wheat

The usefulness of wild species for climate resilience gene sources has been demonstrated most in diploid wild grass *Aegilops tauschii* (genome DD) (Sohail *et al.*, 2011; Elbashir *et al.*, 2017). The reason for this is that the species can be utilized relatively easily by crossing with tetraploid durum (AABB) or bread wheat (AABBDD) to generate newborn wheat, called ‘synthetic wheat’ (Kihara and Lilienfeld, 1949; Chevre *et al.*, 1989; Trethowan and Mujeeb-Kazi, 2008; Zhang *et al.*, 2018). If synthetic wheat is crossed with bread wheat, the genes of synthetic wheat can be transferred to bread wheat without restrictions in meiotic pairing. Synthetic-derived lines, which can be derived from one or more backcrosses to cultivated wheat, can have 10–40% higher yield (del Blanco *et al.*, 2001; Narasimhamoorthy *et al.*, 2006; Trethowan and Mujeeb-Kazi, 2008; W. Yang *et al.*, 2009), including under heat (Cossani and Reynolds, 2015) and drought stress (Lopes and Reynolds, 2011), although the precise genetic bases still need elaboration. At least 85 synthetic-derived wheat varieties have been released around the world since 2003, and evidence for their value is shown in their adoption, such as in India, where synthetic-derived wheat is now grown on >6% of wheat fields (Aberkane *et al.*, 2020).

Other wild relatives also have excellent climate resilience genes and have been indispensable for translocating disease resistance genes in wheat. In some cases, genes from these relatives have resulted in impressive yield gains (Ortiz *et al.*, 2008), such as: the *Thinopyrum* 7E translocation, which showed a 13% increase in yield potential across genetic backgrounds (Reynolds *et al.*, 2001); the *Aegilops ventricosa* 2NS translocation (Juliana *et al.*, 2019); and the rye 1RS translocation, which occupied >50% of CIMMYT breeding lines in the 1980s (Braun *et al.*, 1998; Singh *et al.*, 1998; Sharma *et al.*, 2009). The 1RS translocation has shown positive effects for drought tolerance in various studies using near isogenic lines (Singh *et al.*, 1998; Villareal *et al.*, 1998; Zarco-Hernandez *et al.*, 2005; Hoffmann, 2008) and recombinant inbred lines (Schlegel and Meinel, 1994; Villareal *et al.*, 1995), and induces higher root biomass (Ehdaie *et al.*, 2003; Hoffmann 2008; Sharma *et al.*, 2018) which is advantageous under drought- and heat-stressed conditions (Ludlow and Muchow, 1990; Pinto and Reynolds, 2015). However, in some genetic studies, 1RS decreased grain yield in drought environments (Peake *et al.*, 2011; Tahmasebi *et al.*, 2015), revealing that effects of 1RS will depend on the environment and genetic background. Overall, several translocation lines have been developed but have not been used in wheat breeding due to their agronomically undesirable background (Friebe *et al.*, 1996; Kishii, 2019; Hao *et al.*, 2020). Therefore, it is advantageous to change the background of these existing translocations through backcrossing. and to see their benefits under abiotic stress.

Less than 10% of wild relatives collected have presumably been used in interspecific crossing (Friebe *et al.*, 1996; Kishii, 2019; Hao *et al.*, 2020), and fewer have been surveyed for genetic diversity of traits with potential to boost yield or climate resilience. In addition to developing synthetic hexaploid wheats, to help explore new sources of resilience, new amphiploids can be made (i.e. new hybrids with a diploid set of chromosomes from each parental species) using any *Aegilops* species (the closest genus to wheat). In addition, as a century-old technology, now accelerated by marker technology (King *et al.*, 2017), cytogenetics is a highly effective and non-controversial approach for moving genes between related species to develop improved lines.

An important outcome of crossing with novel sources of stress-adaptive traits, apart from the availability of novel combinations of traits and alleles with potential for direct expression, is the potential for favourable epistatic effects when introduced into local elite backgrounds (though despite the source germplasm having a good agronomic type, the possibility of unfavourable epistatic effects cannot be excluded).

### Improve phenomic tools to identify complementary parental sources, and enrich favourable allele frequencies in selected progeny

Phenotyping is a cornerstone of plant breeding, and effective use of genomic technologies is rooted in the quality and the relevance of phenotyping. To accelerate genetic gain, especially with respect to designing crosses more deterministically and making use of untested genetic resources, genetic and physiological understanding must be underpinned by rigorous phenotyping (Rebetzke *et al.*, 2018; Molero *et al.*, 2019; Reynolds *et al.*, 2020).

**Fig. 7.**
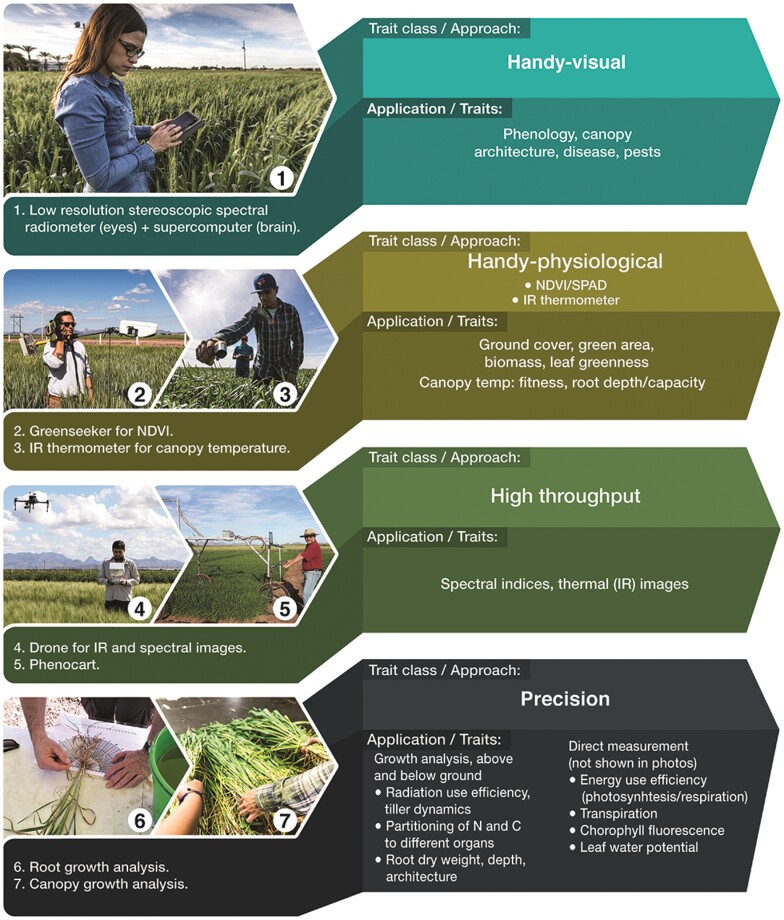
Examples of different classes and applications of breeder-friendly phenotyping (adapted from Reynolds *et al.*, 2020). Abbreviations: NVDI, normalized difference vegetation index; SPAD, a chlorophyll meter. Reprinted under licence CC BY-NC-ND.

The most accessible source of genetic variation for many stress-adaptive traits is within current breeding material. Interestingly, detailed physiological (Crain *et al.*, 2018) and genetic dissection (Rai *et al.*, 2018; Varshney *et al.*, 2018) is not yet a routine procedure for selecting parents among advanced breeding lines. However, developments in field phenotyping now make the selection of adaptive traits feasible at a breeding scale and remove potential subjectivity of reliance on visual scores (Fig. 7).

While these traits, like yield, are also subject to GEI, they can add value to breeding for targeted environments (Richards, 2006; Reynolds and Langridge, 2016; Hunt *et al.*, 2018). This is evident from the major investments that public and private breeding programmes have made in high-throughput phenotyping (HTP) with the expectation of increasing efficiency and selection accuracy (Gaffney *et al.*, 2015; Roitsch *et al.*, 2019). The power of HTP is largely a function of proximal and remote sensing technologies which can measure crop characteristics throughout the season using spectral reflectance or emission, in a non-obtrusive way and at a breeding scale (Araus and Cairns, 2014; Araus and Kefauver, 2018). Furthermore, precision phenotyping of traits that cannot be estimated at high throughput is also becoming more common (e.g. Molero *et al.*, 2019) since their value in designing strategic crosses is recognized (Richards *et al.*, 2010; Reynolds and Langridge, 2016; Reynolds *et al.*, 2020)

There has been an explosion in field phenotyping technologies in recent years (Araus and Cairns, 2014; Araus and Kefauver, 2018), but relatively few have been validated in a breeding and pre-breeding context. Remote sensing can now be applied routinely to measure expression of stress-adaptive traits such as early vigour, cool canopies, estimates of in-season and final biomass, as well as pigments and hydration status that can be estimated with different spectral reflectance indices (Table 2). Other sensor-based approaches, such as RGB imagery or LiDAR, have the capacity to estimate canopy structural traits that can be used to perform spatially and temporally resolved growth analysis, including estimation of phenology. However, for a functional pipeline to be implemented, clearer protocols must be established—including precise phenotyping protocols that consider time of day and crop growth stage, meteorological conditions during data collection, planting method, and the selection environment—that provide the best discrimination of relevant traits for different classes of germplasm at the respective stages of the translational research and breeding pipeline (Reynolds *et al.*, 2020).

**Table 2.** List of remote sensing approaches for high-throughput phenotyping of key adaptive traits for drought and heat in wheat

Trait	Value of trait	Tool/protocol	Index	Reference(s)
*Physiological adaptation*				
Canopy temperature	Indirect estimation of gas exchange rate under heat/drought stress and prediction of root capacity	Handheld IR thermometer, thermography	CT	Amani *et al.* (1996); Lopes and Reynolds (2010); Pinto and Reynolds (2015)
Hydration status	Estimation of soil water access and water relations	Handheld IR thermometer, thermography, spectroscopy	CT, WRI	Gutierrez *et al*, (2010); Pinto and Reynolds (2015)
Photoprotection and photosynthesis	Estimation of potential and actual photosynthetic yield	Spectroscopy, SIF, active fluorometry	PRI, full-spectrum regression models, chlorophyll fluorescence	Pinto *et al.* (2016); Silva-Perez *et al.* (2018)
*Structural and growth dynamics*				
Canopy early vigour	Fast increase of light interception, conservation of soil moisture	Spectroscopy, LiDAR, digital image analysis	NDVI, plant height, leaf area, point cloud density	Mullan and Reynolds (2010); Kipp *et al.* (2014)
Leaf and canopy pigments	Light absorption and photosynthetic activity	Spectroscopy, SPADmeter	NDVI, EVI, CRI, and other SRIs; full-spectrum regression models	Araus *et al.* (2001); Jørgensen *et al.* (2003); Mullan and Mullan (2012); Pietragalla *et al.* (2012)
Stay-green	Prolongs photosynthesis	Spectroscopy	NDVI, EVI, and other chlorophyll-related SRIss	Lopes and Reynolds (2012)
In-season and final biomass; yield estimate	Component of RUE	Spectroscopy, LiDAR, digital image analysis	NDVI, WRI, point cloud density	Babar *et al.* (2006); Jimenez-Berni *et al.* (2018); Walter *et al.* (2018)
Light interception	Component of RUE	Spectroscopy, digital image analysis	NDVI, fraction of intercepted PAR	Liu *et al.* (2013)
Spike counting	Estimation of yield	Digital image analysis	Number of spikes	Deery *et al.* (2014)

CT, canopy temperature; CRI, carotenoid reflectance index; EVI, enhanced vegetative index; LiDAR, light detection and ranging; NDVI, normalized difference reflectance index; PRI, photochemical reflectance index; RUE, radiation use efficiency; SIF, sun-induced chlorophyll fluorescence; SRI, spectral reflectance index; WRI, water reflectance index.

Among the most important traits for adapting to heat and drought are root vigour and depth (Pinto and Reynolds, 2015). Although there have been great advances in the development of high-throughput root phenotyping methods based on imaging techniques, these are usually applicable only under controlled conditions. To date, the only reliable screening technique under field conditions—with limited throughput capacity—is still digging out roots for visual inspection (i.e. shovelomics, York, 2018) or through the quantification of plant DNA extracted from soil cores (Huang *et al.*, 2013).

### Developing a novel selection index based on remote-sensed and stable isotope data to predict root characteristics under stress

The lack of a field-compatible high-throughput screening protocol for root capacity currently precludes any serious consideration of this characteristic in breeding populations or large-scale mining of genetic resources. However, based on a few precedents (Lopes and Reynolds, 2010; Pinto and Reynolds, 2015), there is reason to believe that remote sensing techniques could be developed that predict root capacity. Association between canopy temperature (CT) and root mass has been shown under drought- and heat-stressed conditions, along with genetic bases (Pinto and Reynolds, 2015). This precedent indicates the feasibility of developing a root screening protocol based on remotely sensed traits that could lead to the establishment of a ‘root index’, potentially revolutionizing the ease with which selection for root characteristics could be applied in mainstream breeding of wheat and other crops.

In addition to canopy temperature, other complementary traits can be used to develop such an index, such as the spectrally measured water index (WI) which is sensitive to water fluxes in the canopy (Gutierrez *et al.*, 2010); and carbon isotope discrimination (CID) of grain tissue, which gives an integrated signal of stomatal conductance throughout the grain-filling period (Farquhar *et al.*, 1989) and, in combination with oxygen isotope enrichment (Δ ^18^O) provides additional information about evapotranspiration (Cabrera-Bosquet *et al.*, 2009). Moreover, a close relationship between WI and shoot biomass, a trait that is necessary to consider when evaluating actual root capacity, has been demonstrated (Babar *et al.*, 2006). By measuring CT and WI on leaf canopies under a range of vapour pressure deficits (VPDs) and soil water conditions, and modelling these along with the CID and Δ ^18^O data from grain with actual root and above-ground biomass, we expect to be able to identify the degree to which root capacity can be predicted under varied environmental and management conditions. All this information can be refined into a root index: a non-destructive, HTP, and statistical protocol for estimating root capacity. This would involve machine learning approaches available to determine which remote-sensed indices predict root mass and depth profiles, thereby calibrating the model for ‘root index’ under different stress profiles. If successful, the results are likely to have widespread benefits to other crops and root research in general.

### Determine effects of heat and drought stress on rhizosphere microbiome and its association with genotype adaptation

Both the plant genotype and the environment have strong effects on the composition and diversity of the soil microbiome (Latz *et al.*, 2021). Genotypic traits such as root architecture, turnover, and exudate composition—which vary widely within a species—also directly affect the population size and composition of the rhizospheric microbiota (Schweitzer *et al.*, 2008; Sasse *et al.*, 2018).

Plant growth-promoting rhizobacteria (PGPRs) have been shown to enhance plant resilience to stress (Kumar *et al.*, 2019), and there is considerable interest in unravelling the role of rhizosphere microbiota in crop performance, such as nutrient uptake in wheat (J. Yang *et al.*, 2009; Donn *et al.*, 2015; Azarbad *et al.*, 2018), and on sustainable cropping systems (Ahkami *et al.*, 2017), based on the idea that plants showing drought and heat tolerance will benefit from a self-influenced microbiome that supports adaptation to stress events. For instance, recent findings suggest that drought-stressed plants alter their composition of root exudates to increase microbial activity (de Vries *et al.*, 2019). In return, this may create plant-induced post-drought favourable conditions for plant regrowth, for example for enhanced nutrient availability. Consequently, it can be assumed that particular genotypes with altered post-drought event exudation profiles create a harnessed microbiome for improved drought stress tolerance (de Vries *et al.*, 2020). However, it is unknown whether some genotypes benefit more under drought and heat stress from certain plant genotype-dependent effects on the rhizospheric microbiota which would be potentially important in breeding. While it has been discovered that the genotypic host plant effect on phyllosphere fungal communities of wheat was significant (Sapkota *et al.*, 2015), comparable studies for rhizobacteria are lacking. In cases where rhizobacterial communities can be linked to certain wheat genotypes, PGPRs can also be used to produce plant inoculants to promote plant stress tolerance (Bradáčová *et al.*, 2019; Hafez *et al.*, 2019).

### Elucidate genetic and mechanistic bases of climate resilience to accelerate genetic gain

Stress-adaptive characteristics such as high radiation use efficiency under heat stress, the ability to access subsoil water under drought, and other agronomic traits measured on well-controlled—in terms of height and phenology—but otherwise genetically diverse panels, provide valuable data sets for genetic studies. Such panels include genome-wide association study (GWAS) panels, nested association mapping panels, genetic diversity panels, and trait panels. Known and novel genomic regions related to heat and drought adaptation can be described and summarized in a comprehensive quantitative trait locus (QTL) catalogue. To accomplish this, genomic regions identified across different mapping studies are aligned to available wheat reference genomes or pan-genomes, allowing comparisons across studies at a physical position level. Through review of these comparisons, one can then identify clusters or hot-spot segments harbouring stress-related marker associations, which suggests that they play an important and consistent role across different genetic backgrounds and environments. Results can include or be compared with meta-analyses studies (Griffiths *et al.*, 2009; Acuña-Galindo *et al.*, 2015; Soriano and Alvaro, 2019). Meta-analyses across GWAS can be applied which use summary statistics across multiple GWAS data sets to calculate a global *P*-value considering diverse environments and trials. Due to a larger sample size, SNP effect estimates (and their SEs) from multiple experiments including unbalanced phenotypes used in a single meta-analysis have been shown to result in higher power and fewer false positives (Evangelou and Ioannidis, 2013; Joukhadar *et al.*, 2021).

The newest genomics tools include access to fully annotated and ordered wheat genome sequences from the International Wheat Genome Sequencing Consortium (RefSeq v2.0, http://www.wheatgenome.org/) and the ‘pan genome’ from the 10+ Wheat Genomes Project (Montenegro *et al.*, 2017), which will locate markers and determine the structures (introns, exons, promoter, and untranslated regions) faster and with greater accuracy than before. Third-generation sequencing data of 100 key CIMMYT breeding lines are becoming available to explore and use, in addition to high-quality assemblies with large genome coverage of another eight CIMMYT wheat lines known for specific stress adaptation.

The above cross-cutting germplasm and genomic resources will serve as crucial adjuncts to variant interpretation, allowing a new way of allele mining and search for high-value functional variants. In this review, the potential of epigenetics is not covered since translation will require a larger knowledge base than currently available (Varotto *et al.*, 2020). Similarly, gene editing is not discussed due to the sparsity of cloned genes validated for improving heat and drought stress adaptation in the field as well as the uncertainty of if, or how, new genomics technologies (such as CRISPR) will be regulated in some countries/regions, such as the European Union [EFSA (European Food Safety Authority) *et al.*, 2021]—a question that has hampered mainstream integration of such technologies into internationally focused breeding programmes.

### Predict the performance and determine the genetic bases of climate resilience associated with the wild species (D genome) introduced into synthetic wheat

Synthetic wheat provides totally new genomes for use in breeding and has already been shown to contribute both biotic and abiotic stress adaptation, as evidenced by the pedigrees of many modern wheat varieties (Dreisigacker *et al.*, 2008; Rosyara *et al.*, 2019). Since the 1980s, CIMMYT has developed >1500 synthetic wheat lines (Rosyara *et al.*, 2019). Marker-based estimates indicate that 20% of the lines in CIMMYT’s bread wheat programme international nurseries are synthetic derived, with the D genome (from *A. tauschii*) contributing to 15.6% of them (Rosyara *et al.*, 2019). There is clear evidence that many of these synthetic-derived wheat varieties express superior fitness under heat and/or drought stress (Lopes and Reynolds, 2011; Cossani and Reynolds, 2015; Reynolds *et al.*, 2017*b*), but their full genetic potential has yet to be exploited (Reynolds *et al.*, 2015), due to difficulty predicting the performance of synthetic wheat in the bread wheat background. Furthermore, linkage drag from the less domesticated part of the D genome in synthetic wheat lines reduces the efficient introgression of novel genomic regions during selection (Voss-Fels *et al.*, 2017).

Genomics-assisted approaches can be used to address some of these questions. The potential of synthetic wheat as parents in crosses can be estimated using prediction strategies similar to hybrid wheat (Basnet *et al.*, 2019). In addition, a novel approach can be employed to pinpoint regions specific to the D genome (the main bottleneck in modern hexaploids) by crossing synthetic wheat lines such that only the wild D genome shows segregation among the progeny, permitting more effective genetic analysis and marker development. In this approach, primary synthetic lines having the same AB genome but different D genomes are crossed to develop multiple recombinant inbred line populations.

### Going beyond the genes: metabolomic studies to identify mechanistic and genetic bases of climate resilience traits

More than 95% of crop biomass dry matter is derived from photosynthate (Makino, 2011), of which >95% is derived directly from photosynthetic sugars. The enormous complexity of intermediary metabolism makes it nearly impossible to pinpoint the limiting steps in photosynthate production.

Photosynthetic metabolites are the final products of intermediary metabolism preceding plant biomass formation. Thus, instead of making *a priori* assumptions as to which metabolic step to alter to increase photosynthate production and transport to the developing grain, the components of total photosynthate [including simple sugars (fructose, glucose, and sucrose), fructan (a polymer of fructosyl residues), and leaf starch] can be screened. In addition, although the whole-plant metabolome can be measured (Riedelsheimer *et al.*, 2012; Hill *et al.*, 2013), throughput remains a problem. Instead, high-throughput, 96-well assays can precisely measure various photosynthate components and provide abundant precise data to elucidate the main bottleneck to yield under heat and drought stress: photosynthate. High-throughput assays for various metabolites and enzymes have been successfully used previously to screen mapping populations (Silva *et al.*, 2017, 2018).

Approximately 10–12 d between flowering and the log phase of grain filling, canopy photosynthate production probably exceeds the demand from secondary growth (Wheeler *et al.*, 1996). In the absence of an active sink, surplus photosynthate is stored as fructan, mainly in the culm but also in the leaves for later use in grain development. Some photosynthate is also stored as starch in the chloroplasts during the day and recycled at night to support respiration as well as secondary growth. The lag phase of grain development is ideal for tissue sampling (e.g. culm, leaves, leaf sheaths, and spike) to measure the plant’s potential to produce photosynthate in the absence of a strong sink (e.g. developing grain). Culm tissue can be collected, again, at physiological maturity to determine the remobilization efficiency of the fructans and their buffering capacity against drought and heat stress.

Identifying genetic variation for photosynthate production, particularly under drought and heat stress, would provide additional tools to break the currently stagnant rate of yield improvement. Additionally, association mapping of the various photosynthetic components can lead to the identification of markers and candidate genes. All of these tools would be powerful resources to assist breeding programmes (Guo *et al.*, 2018; Thomason *et al.*, 2018).

### Refining methods to incorporate new sources of heat and drought stress resistance into adapted backgrounds

**Fig. 8.**
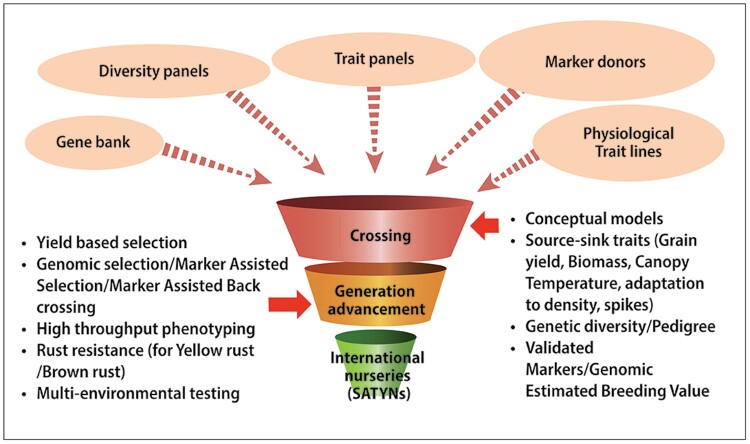
Pre-breeding pipeline incorporating diverse genetic resources into elite widely adapted materials and delivering semi-elite high-value germplasm as the stress adaptive trait yield nurseries (SATYNs) to countries around the world.

Current yield gains in wheat result mainly from unspecified recombination of genes of minor effect among elite germplasm, in other words ‘crossing best×best and selecting the best’ (van Ginkel and Ortiz, 2018). In mainstream breeding, introduction of novel genetic diversity is typically driven by the need for disease resistance or grain quality traits. In wheat, several diagnostic markers are routinely used, including *Vrn*, *Ppd*, *Rht*, and major disease resistance genes (Dreisigacker *et al.*, 2016), using backcrossing and marker-assisted selection for major genes (Collard and Mackill, 2008). Marker-assisted selection for complex physiological traits with underlying small-effect genes remains a challenge. Nonetheless, evidence has been accumulating that deterministic selection for complex traits to boost yield can be achieved through phenomic- and genomic-assisted breeding, where a diverse set of genetic resources are used to cross with elite lines. These segregating generations undergo selection based on physiological and genetic screening approaches to deliver high-value semi-elite materials (Fig. 8).

Phenomics-assisted breeding consists of detailed measurement of physiological traits—through ground-based and remote sensing-based approaches (Fig. 7)—to identify parental sources encompassing potentially complementary traits as well as superior progeny (Reynolds *et al.*, 2020). Crossing with and selection for traits such as biomass (associated with radiation use efficiency and efficient use of water under heat and water stress, respectively) and cooler canopies (associated with more extensive root systems) have resulted in some outstanding progeny (Reynolds and Langridge, 2016; Reynolds *et al.*, 2017*b*) including novel varietal releases in climate-challenged regions in South Asia (Reynolds 2019).

**Fig. 9.**
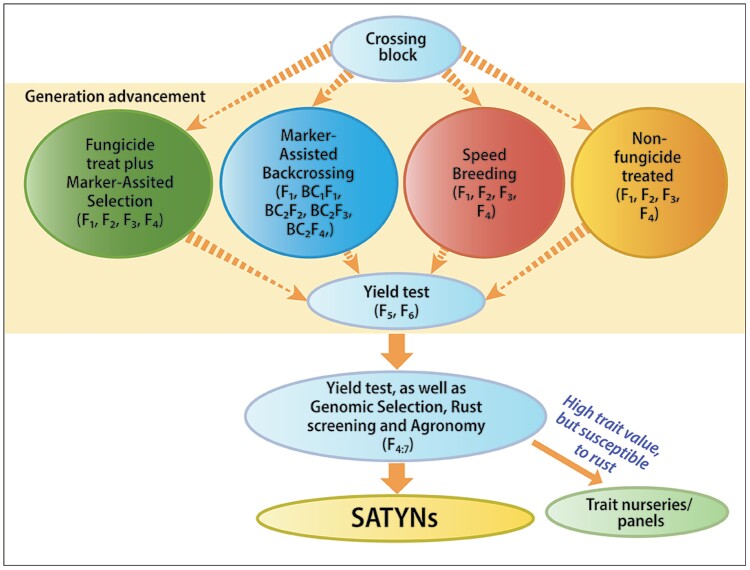
Different streams of a pre-breeding pipeline for spring wheat breeding at CIMMYT, including selection with and without fungicide treatment, marker-assisted backcrossing, and speed breeding. Lines undergo genomic selection and rust screening, and are further examined for agronomic traits routinely at the F_4:7_ stage, though these examinations may occur in earlier generations depending on the model and needs. Lines with good trait values as well as rust resistance are included in the stress adaptive trait yield nurseries (SATYNs), for global distribution through the IWIN, while those that have good trait value, but are susceptible to rust, are recycled into the programme through trait nurseries and germplasm panels used in crossing.

Genomics-assisted breeding has the potential to accelerate genetic gain through approaches such as marker-assisted selection, marker-assisted backcrossing, and genomic selection (GS; Varshney *et al.*, 2018, 2020). While these approaches have been used and are largely expanded in mainstream breeding, they must also be explored when focusing on novel genes identified from genetic resources such as resynthesized hexaploid wheat (synthetic wheat) with potentially large effects (Afzal *et al.*, 2019). These novel genes are paramount to allow further breeding progress. However, exact pre-breeding methods for quantitative traits are not well established. Incorporating novel traits and genes from unadapted germplasm to elite germplasm is a challenge, due to potential linkage drag, the complexity of trait stacking and phenotyping, and a lack of validated selection strategies. In addition, breeders are quite conservative about utilizing pre-bred material as sources of stress resistance, unless also encompassing desired agronomic traits such as acceptable height and maturity range, and disease resistance, as there is the potential to break up useful linkage blocks present in elite breeding germplasm. Two key questions that need to be addressed are (i) whether selection for heat- and drought-adaptive traits will be more successful if it is not confounded by simultaneous selection for rust resistance (assuming that it is more cost-effective to incorporate disease resistance at a later stage through backcrossing)—this can be tested relatively simply with and without inoculation to create disease pressure in segregating generations; and (ii) whether rapid generation advancement (RGA) in the greenhouse can accelerate the incorporation of desirable traits without a significant negative impact on the agronomic characteristics of progeny (Fig. 9). Since, ideally, pre-breeding achieves rapid proofs of concept regarding the value of new trait combinations, the potential to accelerate pre-breeding by applying SpeedGS (i.e. integrated speed breeding, a method of RGA where plants can be grown in a temperature-controlled glasshouse under a prolonged photoperiod to increase the rate of development, along with GS) is important (Watson *et al.*, 2018; Voss-Fels *et al.*, 2019*b*).

The combined use of phenomics and genomics is already showing itself to be a powerful approach in pre-breeding for yield potential under the IWYP platform (Reynolds, 2019). In recent years, breeding programmes have started to implement GS, but this has been primarily focused on improving genomic prediction accuracies using different cross-validation models (Crossa *et al.*, 2017), such as those incorporating reaction norms (Jarquín *et al.*, 2014) and GEI (Sukumaran *et al.*, 2018), combined with pedigree-based breeding. The main advantage of GS over phenotypic selection for yield *per se* is that it can expedite the selection of superior genotypes by allowing prediction of performance in generations×locations, in cases where phenotyping cannot be directly conducted (e.g. sparse testing), thus reducing phenotyping costs and accelerating breeding cycles. Simulations have shown that SpeedGS in combination with phenomics can accelerate genetic gain per year and facilitate a more rapid incorporation of novel genetic variation into breeding programmes, compared with pedigree-based breeding (Voss-Fels *et al.*, 2019*a*, *b*). In essence, phenomics, for example measurements of yield secondary traits on selection candidates, can be incorporated into multivariate GS models together with field-measured grain yield of a training population with the aim of increasing the prediction ability above that of a univariate genomic selection approach (Watson *et al.*, 2019).

### Create novel germplasm using spring×winter wheat crosses to accumulate beneficial alleles from isolated elite genepools

Crossing spring with winter germplasm has resulted in a number of successes in the past, in terms of transferring new sources of disease resistance and boosting yield potential and other important traits in both cultivars. For example, the rye 1RS translocation described in an earlier section was derived from such a crossing programme impacting both spring and winter CIMMYT breeding lines (Braun *et al.*, 1998; Singh *et al.*, 1998; Sharma *et al.*, 2009). Among many, ‘Veery’ and its progenies, such as ‘Kauz’, ‘Attila’, ‘Pastor’, and ‘Baviacora’, were the most successful varieties developed through a large-scale spring×winter wheat crossing programme at CIMMYT (Rajaram and van Ginkel, 1996). Rajaram and van Ginkel (1996) further observed that most of the spring×winter wheat crosses resulted in vigorous progenies with profuse tillers, robust spikes, and healthy leaves with prolonged activity.

Although former crossing efforts had great success, the genetic and physiological bases of benefits to abiotic stress were not examined. In addition, although there is a reason to believe that alleles for abiotic stress resilience genetics is different between spring and winter genepools, to our knowledge, the opportunity to boost climate resilience by combining the best sources of resilience between the spring and winter wheat genepools is not being pursued extensively. In a study by Larsen (2012), it was suggested that winter×spring crosses can be effectively utilized to develop winter-hardy spring wheat germplasm. Conversely, Tsenov *et al.* (2016) reported that it is possible to develop early-flowering and early-maturing winter wheat germplasm by crossing with the spring type and maintaining the cold tolerance through selection in appropriate environments. Moreover, some of the cold tolerance genes, such as FR-2, are found to be associated with C-repeat binding factors (CBFs) which induce expression of a series of genes that enhances the tolerance to cold and drought stress in plants (Thomashow, 2001; Miller *et al.*, 2006; Knox *et al.*, 2008). The possibility that common genetic factors are responsible for both cold and drought tolerance has been reported in several eminent studies in the past (Liu *et al.*, 1998; Kasuga *et al.*, 1999). Based on past research evidence and current knowledge of performance of both spring and winter wheat under heat- and drought-stressed environments, strategic crosses can be made between spring and winter lines with the goal of combining climate resilience traits and alleles into useful agronomic backgrounds. The progeny would comprise both spring and winter types for use by breeders worldwide. Thus, although the specific benefits to abiotic stress remain to be determined, outputs would establish the potential value of combining elite genetic backgrounds between these mostly isolated genepools and whether this represents a viable method for accelerating climate resilience breeding.

### Continuous improvement of breeding wheat for climate resilience

CIMMYT’s research and breeding activities have driven variety improvement globally via the IWIN nurseries (Braun *et al.*, 2010; Lantican *et al.*, 2016; Reynolds *et al.*, 2017*a*). Throughout the years, the CIMMYT wheat breeding programmes have continually modified strategies and methods to best serve the principal targeted wheat-growing countries in Asia, Africa, and Latin America, improving grain yield potential and stability combined with climate resilience, disease resistance, and appropriate end-use quality, with considerable returns on investment and a well-documented impact (Lantican *et al.*, 2016). Periodically estimated genetic gain in grain yield in the CIMMYT spring bread wheat programme varies from 0.6% to 1.1% (Crespo-Herrera *et al.*, 2017, 2018; Mondal *et al.*, 2020). To accelerate varietal development and population improvement in the currently changing climate scenario, CIMMYT is looking forward to piloting and adopting new breeding approaches, which combine advanced genomics and phenomics, as well as a reduced breeding cycle time to increase rates of genetic gains.

### Use of genomic-estimated breeding values in parent selection and generation advance

The design of new crosses is one of the most important decisions to be made in a breeding programme. The decision of which lines to cross is, in most plant breeding programmes, mainly based on aggregated phenotypic data of potential parents. Deploying additive genetic values has a long history in animal breeding, where classical family or pedigree relationships and phenotypic data are used to estimate breeding values (Henderson, 1975). The additive genetic value or the breeding value of lines derived from pedigrees has been used at CIMMYT, but not for the analyses of all existing trials. With the availability of genotypic data, the realized genetic relationships derived from genome-wide markers is used for predicting breeding values of lines (Meuwissen *et al.*, 2001; Crossa *et al.*, 2010). These genomic-estimated breeding values (GEBVs) together with the pedigree and phenotypic information will enable breeders to make informed decisions on which individuals to use in crossing and select simultaneously on several desired superior alleles, which have the potential to increase the efficiency of selecting the best performers and also facilitate discarding the poor performers (Heffner *et al.*, 2009).

Recognizing the clear need to complement phenotypic data-based parental selection with additional data that can improve precision, holistic data-driven approaches leveraging all the existing phenotypic, pedigree, and genomic data must be used for identifying the best parents with the highest breeding values for key traits. This would involve developing and validating computationally efficient statistical models incorporating all the available information for a line to estimate the breeding values and integrating them with phenotypic data to select high-value parents for grain yield and stress resilience. Furthermore, machine learning methods hold great promise for predicting the breeding values of complex traits (Montesinos-López *et al.*, 2018*a*, 2019; Crossa *et al.*, 2019), and a variety of deep learning methods can be explored for obtaining GEBVs.

In addition to selecting the best parents, predicting crosses that have the highest likelihood to result in superior progenies is critical to increasing the breeding efficiency and genetic gains. Cross-prediction can be based on the mean of the parental GEBVs (Endelman, 2011), the genetic variance in bi-parental breeding populations (Mohammadi *et al.*, 2015), combinations of mid-parent value and variance predictions (Lado *et al.*, 2017), and other inputs.

### A rapid generation advancement and/or a genomic selection-assisted breeding scheme

There has been continuous improvement in wheat breeding by testing and adopting new approaches to increase breeding efficiency and genetic gain (Voss-fels *et al.*, 2019). In recent years, several new breeding concepts combining genomics and RGA have been discussed in the literature that could potentially increase the rates of genetic gain in breeding programmes including for wheat (Atlin *et al.*, 2017; Gaynor *et al.*, 2017; Voss-Fels *et al.*, 2019*a*; Watson *et al.*, 2019). For example, Gaynor *et al.* (2017) suggested a two-part breeding strategy that reorganizes a breeding programme into two distinct components: (i) a population improvement component to identify parents for subsequent breeding cycles and increase the frequency of favourable alleles through rapid recurrent GS; and (ii) a product development component, to develop advanced breeding lines. The population improvement component relies on recurrent selection in an early breeding generation using GEBVs, expected to result in a fast increase of population means.

A second approach to turn generations over more quickly and thus to make faster breeding progress is RGA. Systems have been adapted to fully enclosed growth chambers, as well as glasshouses to facilitate generation advancement for breeding programmes (Ghosh *et al.*, 2018; Watson *et al.*, 2018). This approach can assist breeders to reduce the duration of the breeding cycle and ultimately increase the rate of genetic gain per unit of time.

At CIMMYT, existing genomic and phenotypic data from the spring bread wheat breeding programme are a potential resource for piloting the new genomics- and RGA-based breeding schemes. The aim is to rapidly turn over generations as bulks through RGA and early generation selection using GEBVs for advancing decisions and selection of parents for the subsequent breeding cycle.

### Development of advanced bioinformatics and utilization for predicting breeding values

Improvement in sequencing technologies has enabled the acquisition of high-quality wheat reference genomes, and subsequent re-sequencing efforts will capture genetic variation present in elite germplasm pools at the genome-wide level. Computational technologies, advanced bioinformatic tools, and databases capitalizing on the available whole-genome sequence data are, however, still limited in plant breeding programmes. Currently, next-generation sequencing reads from CIMMYT wheat breeding lines are aligned to the reference genome of Chinese Spring (genetically detached from CIMMYT’s elite gene pool), which most probably impairs genetic analysis such as the prediction of GEBVs. Genome catalogues or multigenome reference graphs can capture a wider range of genetic variation and can therefore improve the mapping sensitivity of short sequence reads (Rakocevic *et al.*, 2019). Computational pipelines that can extrapolate from whole-genome sequences and impute more accurate haplotypes of individuals in a breeding population therefore most probably enhance the potential of genomic analyses. CIMMYT had aimed to adopt the computational pipeline (called the Practical Haplotype Graph) recently developed at Cornell University (https://bitbucket.org/bucklerlab/practicalhaplotypegraph/wiki/Home, Jensen *et al.*, 2020), which establishes a multigenome reference. However, adoption has been difficult, given the large genome size of wheat. Therefore, new solutions are still required and need to be integrated in routine genomic prediction analyses for key traits to further increase the accuracy of GEBVs.

### Implement strategies to integrate stress-adapted germplasm through mainstream breeding pipelines for key production regions

Although climate-resilient yield-stable wheat is paramount for food security and facing the uncertainties of the temporal and spatial variations, mainstream breeding pipelines need to develop germplasm that incorporates numerous other traits to meet multiple needs of growers, such as disease resistance and end-use quality traits. Considering this, breeding programmes must strategize the use of the previously described approaches to complement activities to maintain or improve other traits critical to targeted producers and markets production regions. Thus, clear characterization of producer and market needs is paramount to inform strategic crossing and cost-effective selection tools that address multiple traits. Utilization of climate-resilient germplasm donors (from pre-breeding outputs and/or existing elite germplasm) for strategic crossing can be implemented by first carefully choosing the progenitors to use in the mainstream breeding population. Regarding selection and advancement in the breeding programme, cost-effective and breeder-accessible selection tools are fundamental to identify the best progeny derived from the strategic crossing (Reynolds *et al.*, 2020). These selection tools span phenotyping and genotyping technologies as well as advanced statistical methods to predict phenotypic performance (Lopez-Cruz *et al.*, 2015). In addition, multienvironment testing of advanced progeny is fundamental for confirming that wheat germplasm carries a package of desired traits. Multienvironmental testing is one of the cornerstones of wheat breeding at CIMMYT, and through the IWIN it is possible to assess newly developed germplasm globally and utilize the evaluation results to make breeding decisions.

### Validate and disseminate new breeding technologies through the IWIN

New technologies, such as those described in previous sections including new sources of resilience, new pre-breeding approaches, results of genomics and phenomics, etc., as well as other technologies that have not been described (such as epigenetic modifications and gene editing), can be validated through the IWIN, the largest public–private network of wheat collaborators in the world, which routinely tests new bread and durum wheat and triticale lines (Braun *et al.*, 2010; Fig. 2). CIMMYT annually updates and distributes, on request, various sets of international yield trials and observation nurseries targeted to specific mega-environments (Braun *et al.*, 2010; Gbegbelegbe *et al.*, 2017) and biotic stresses (Table 1). While germplasm provided to national and private breeding programmes is used mainly as sources of traits for breeding, and as candidates for variety release, data on adaptive responses of new lines are shared within the IWIN, providing key insight to refine research and breeding methods (Gourdji *et al.*, 2013; Lantican *et al.*, 2016; Crespo-Herrera *et al.*, 2017, 2018). Therefore, the IWIN provides the ideal platform for validating impacts of translational research (Reynolds *et al.*, 2017*b*, 2019) and breeding (Crespo-Herrera *et al.*, 2017, 2018), relying on massive in-kind contributions from its members in both public and private sectors.

### Crowd-sourcing novel plant science technologies to increase impacts on climate resilience of wheat and other crops

To ensure that the translational research and breeding pipelines capitalize on cutting-edge developments in science, promising new ideas need to be tested in a realistic breeding context, opening up bottlenecks between discovery research and wheat breeding. One way to achieve this is through calls aimed at the international plant research community, with the view to accelerate the transfer of technologies from the laboratory to field breeding. Such ideas may encompass: testing novel traits likely to boost climate resilience; methods for accessing a wider range of genetic resources; improving the practical understanding of trait and allelic interactions in successful progeny; and validating new phenomic and genomic selection models. While the immediate benefit would be to wheat, it is expected that useful breakthroughs can be scaled out to other crops. Additional benefits include more efficient use of research resources and opportunities for closer interactions between disciplinary scientists and practising breeders. Testing of crowd-sourced ideas can be supported through links to the IWIN by provision of exotic germplasm, panels of elite breeding materials, and managed stress profiles in actual breeding environments.

### Improved research capacity and technology scale-out

New breeding technologies developed by CIMMYT and collaborators are delivered through the IWIN and other partnerships (see https://wheat.org/download/wheat-phase-ii-full-proposal/), and include a variety of outputs, such as advanced lines and experimental germplasm, research results and other new knowledge, and curated data and metadata sets. Capacity building has also been an important output of the IWIN, sharing knowledge through publications of research results and methodologies; and training activities from short-term practical courses to mentoring of PhD candidates and early career scientists. In addition, and through linkages with the HeDWIC and other initiatives, expertise and research infrastructure can be shared and developed through collaborative agreements among laboratories worldwide, resulting in significant scale-out of capacity, including to other major staple crops and possibly laying the foundation of climate resilience research for a range of other ‘orphan’ crops.

## Conclusions

The explosion in fundamental plant science in recent decades has uncovered the physiological and genetic bases of many traits, as well as genetic markers and assays to select for them. This has resulted in a massive pileup of ideas that have yet to be tested and translated into applied breeding programmes (Jha *et al.*, 2014; Mathan *et al.*, 2016; Govindaraj *et al.*, 2018; Lamaoui *et al.*, 2018; Sreeman *et al.*, 2018); these include candidate traits and genes for climate resilience, use of the wheat genome sequence in gene discovery, and advances in HTP, machine learning, etc. (Araus *et al.*, 2018; Hu *et al.*, 2018; Monroe *et al.*, 2018). In the same time frame, the world’s population has almost doubled, the natural resource base for agricultural productivity is threatened by reduced water supplies and wide-scale soil erosion, and climate is becoming more challenging for agriculture. Clearly, the need for investment in translational research—linking promising ideas to actually improving crop cultivars—is more critical than ever. However, relatively few scientists occupy the applied research space in which proofs of concept for climate resilience technologies are rigorously tested in a breeding context. Therefore, lacking adequate proofs of concept, many proposed technologies with potential impact on crop improvement remain on the shelf, with a few exceptions (Reynolds *et al.*, 2019). This review outlines ideas to link discovery research to breeding for climate resilience in a systematic way (Fig. 4). Since the bottleneck between upstream plant research and crop improvement is widespread, ideas developed herein could also serve other crop programmes facing similar challenges.

## Abbreviations:

CIMMYT: International Maize and Wheat Improvement Center
CT: canopy temperature
GEBV: genomic-estimated breeding values
GEI: genotype by environment interaction
GS: genomic selection
GWAS: genome-wide association study
HeDWIC: Heat and Drought Wheat Improvement Consortium
IWIN: International Wheat Improvement Network
IWYP: International Wheat Yield Partnership
RGA: rapid generation advancement
WI: water index
